# *CLOCK* gene polymorphisms and quality of aging in a cohort of nonagenarians – The MUGELLO Study

**DOI:** 10.1038/s41598-018-37992-8

**Published:** 2019-02-06

**Authors:** Giuditta Pagliai, Francesco Sofi, Monica Dinu, Elena Sticchi, Federica Vannetti, Raffaele Molino Lova, José Marìa Ordovàs, Anna Maria Gori, Rossella Marcucci, Betti Giusti, Claudio Macchi

**Affiliations:** 1 Department of Experimental and Clinical Medicine, University of Florence, Florence, Italy; 2 IRCCS Don Carlo Gnocchi Foundation Italy, Florence, Italy; 3 Atherothrombotic Unit, Careggi University Hospital, Florence, Italy; 4 Jean Mayer US Department of Agriculture Human Nutrition Research Center on Aging, Tufts University School of Medicine, Boston, MA USA

## Abstract

A total of 356 elderly subjects [257F; 88–106 years] were genotyped for three polymorphisms of the CLOCK gene by TaqMan real-time PCR approach, in order to find associations with quality of aging. Subjects homozygous for the minor allele of rs1801260 were less frequently overweight (p = 0.046), had higher fasting glucose levels (p = 0.037), better scores at the Clock Drawing Test (CDT) (p = 0.047) and worse scores at the Geriatric Depression Scale (p = 0.032). Subjects homozygous for the minor allele of rs11932595 showed higher fasting glucose levels (p = 0.044) and better scores at CDT (p = 0.030). Conversely, subjects homozygous for the minor allele of rs4580704 showed higher triglyceride (p = 0.012), and LDL-cholesterol levels (p = 0.44), and a greater adherence to the Mediterranean diet (MD) (p = 0.044). In addition, AAC, AAG, GGC and AGC (rs1801260–rs11932595–rs4580704) haplotypes were analyzed: AAG was associated with higher risk of overweight (p = 0.008), hypertriglyceridemia (p = 0.040) and hypercholesterolemia (p = 0.036); GGC with lower risk of hyperglycemia (p = 0.022), better sleep pattern (p = 0.001) and with better score at mini-mental state examination (p = 0.010); AGC with lower risk of depression (p = 0.026) and AAC with lower adherence to the MD (p = 0.028). Therefore, CLOCK gene polymorphisms let us hypothesize an involvement in the quality of aging in a cohort of nonagenarians.

## Introduction

The age of the population is constantly increasing. Epidemiological studies show that 11% of the world’s population is over 60 years old, with an expected increase, in 2050, to 22%^1^. As aging is often associated with the development of the disease, this leads to an increase in age-related pathological conditions, such as cancer, cardiovascular and neurodegenerative diseases. In addition to environmental and lifestyle factors, it is well known that both genetic and epigenetic aspects play a central role in the development of these diseases^2^.

In recent years, most of the attention has been focused on the genes of the circadian clock, a series of genes involved in maintaining the internal coordination of multiple oscillators within and between various organs systems, in order to increase the physical fitness of an organism and provide the most efficient response to the periodical environmental events such as the day/night cycle^3^. The Circadian Locomotor Output Cycles Kaput (*CLOCK*) gene is one of the first genes studied in this regard: it codifies for the *CLOCK* protein, a positive regulatory arm of the circadian system. The alterations of this complex system – that is shift work, sleep deprivation, exposure to intense light at night, as well as the physiological process of aging – or genetic variation of the *CLOCK* gene, have been associated with several physiological and pathological conditions. In particular, variations of the *CLOCK* gene have been associated with the risk of obesity, type 2 diabetes, sleep and mood disorders, and different types of cancer^4–6^. With regard to aging, few studies have investigated the possible role of the *CLOCK* gene on longevity^7,8^, but to the best of our knowledge, no studies have investigated the possible influence of *CLOCK* gene SNPs on the quality of aging in very elderly subjects.

The aim of the present study was to investigate the relationship between three common polymorphisms known in the *CLOCK* gene and the aging process, in relation to cardiovascular risk factors, quality of sleep, adherence to the Mediterranean diet, as well as cognitive and functional skills, in a cohort of nonagenarians enrolled in the frame of an epidemiological study conducted in the Mugello area, Tuscany, Italy^9^.

## Material and Methods

### Study population

The study population consisted of 356 elderly subjects [257 women, 99 men; median age 92 (88–106) years] enrolled in the Mugello study, an epidemiological study aimed at investigating some clinically relevant geriatric items related to the quality of aging, as well as various health issues including those related to the nutritional status of a group of nonagenarians living in the Mugello area, Tuscany, Italy. The details of the study protocol and the characteristics of the study population have been described elsewhere^9^. The study was conducted according to the Helsinki Declaration on Clinical Research involving Human Subjects and was approved by the Ethics Committee of the Don Gnocchi Foundation. All participants, or their legal representative, have signed the informed consent form.

### Data collection and measurements

#### Demographic characteristics

The subjects enrolled were evaluated during home visits/nursing home, through objective examinations and questionnaires regarding lifestyle, dietary habits and cognitive status. General information on demographics, education, personal medical history and drug use was collected by each participant. After the subjects were rested for 5 minutes in a quiet room, arterial systolic and diastolic blood pressure were measured on the right arm with a standard mercury sphygmomanometer while the subjects were sitting in a sitting position. After 12 hours of nocturnal fasting, venous blood samples were obtained from participants. Laboratory parameters were analyzed using standard procedures.

#### Anthropometric measurements

Anthropometric data, such as weight, height and waist circumference were collected by the participants. The body mass index (BMI) was calculated as the weight in kilograms divided by height in square metres. The classification of the World Health Organization for the categories of BMI, in terms of underweight (BMI < 18.5), normal weight (BMI = 18.5–24.9), overweight (BMI = 25.0–29.9) and obese (BMI ≥ 30) was used to classify the patients.

#### Sleep pattern determination

The sleep pattern of patients was assessed using the Pittsburgh Sleep Quality Index (PSQI)^10^, which is a validated self-assessed questionnaire comprising nineteen individual elements that generate seven scores related to sleep quality, sleep latency, sleep duration, habitual sleep efficiency, sleep disturbances, use of sleep medications, and daytime dysfunction. The score of the answers is based on a scale from 0 to 3, in which 3 reflects the extreme negative. A global sum of “5” or greater indicates a “poor” sleeper. The sleep pattern information was also collected by using a SenseWear Armband calorimeter (BodyMedia Inc, USA), applied to the participants over the triceps muscle, halfway between the acromion and the olecranon processes, as suggested by the manufacturer. The participants were asked to wear ArmBand for at least a week, removing it only for bathing or showering.

#### Cognitive and functional abilities determination

Regarding cognitive skills, a shorter version of the Geriatric Depression Scale (GDS)^11^, consisting of 15 items, was used to detect the possible presence of depressive symptoms. Scores ≥5 were considered indicative of depression. The cognitive impairment comprising the memory, orientation, attention and language of the participants was evaluated by the Mini Mental State Examination (MMSE)^12^: the overall score ranged from 0 and 30 and a score of 23 or less was used to indicate cognitive impairment. In addition, a Clock Drawing Test (CDT)^13^ was used, with a global score ranging from 0 (without cognitive impairment) to 10 (prominent cognitive impairment), to investigate visuo-constructional skills and abstract thinking. Functional ability was assessed using the Basic Activities of Daily Living (BADL)^14^ – with a total score ranging from 0 (independence in all functions) to 6 (complete dependence) – and the Instrumental Activities of Daily Living (IADL)^15^ – with a total score between 0 (complete independence) to 8 (complete dependence). Finally, the Frailty Index 34 (FI-34)^16^ was used to measure the health status of older individuals using a series of 34 aging-related variables as a substitute for aging and vulnerability to negative outcomes: the overall score ranged from 0 (no frailty) to 1 (prominent frailty).

#### Dietary evaluation

The dietary intake was evaluated through the Mediterranean Diet Score, devised by Panagiotakos *et al*.^17^. The questionnaire consisted of 11 items, with a global score ranging from 0, for a minimal adherence to the traditional Mediterranean Diet, to 55 for maximum adherence.

### *CLOCK* Genotyping

We selected tag single nucleotide polymorphisms (tag SNPs) as effective proxies for untyped SNPs in strong linkage disequilibrium (LD) using the Tagger-pairwise Tagging algorithm (HapMap database and software available at http://hapmap.ncbi.nlm.nih.gov) on the *CLOCK* gene based on Caucasian European Utah data with a minor allele frequency (MAF) ≥0.20 and a minimum r^2^ of 0.8. The following SNPs have been selected: rs3736544, rs11932595 and rs17722979. Based on information on linkage disequilibrium and previous reports showing associations with cardio-metabolic outcomes, rs17722979 was replaced by rs1801260 (known as 3111T/C), and rs3736544 was replaced by rs4580704. For haplotype analysis, we estimated haplotype frequencies using expectation-maximization algorithm implemented in HelixTree software (GOLDEN Helix, Bozeman, MN).

DNA was isolated from blood samples using Flexigene DNA kit (Qiagen, Hilden, Germany). We performed genotyping of *CLOCK* gene polymorphisms using a TaqMan assay with allele-specific probes (ThermoFisher Scientific) on the ABI Prism 7900HT Sequence Detection System (Applied Biosystem, Foster City, CA, USA) according to the standardized laboratory protocol^18^.

### Statistical analysis

Statistical analysis was performed using the SPSS (Chicago, IL) software for Macintosh (version 19.0). Values are expressed as mean and 95% Confidence Interval (CI). Log-transformed values for all the parameters were used in the analyses and back transformed for data presentation. Different genetic inherent models were tested, and a recessive model was applied in the final analyses for all the selected SNPs. The chi-square test was used to test for deviations of genotype distributions from the Hardy–Weinberg equilibrium (HWE), to detect any differences in genotype distributions based on sex and to detect any differences in proportions – e.g. sex distribution, categories of BMI and adherence to the Mediterranean Diet – according to genotype. The associations between the different genotypes and the general characteristics of the study population was tested using the analysis of variance (ANOVA) adjusted for all the possible confounding factors. Furthermore, in order to estimate the association between haplotypes and all the variables tested, a logistic regression analysis was performed adjusted for age, sex, BMI (as continuous variable) and specific medication (statins for lipid parameters; beta-blockers, diuretics, calcium antagonists and ACE-inhibitors for blood pressure; anti-diabetic drugs and insulin for blood glucose; antidepressants for depression, and benzodiazepines for cognitive parameters and sleep pattern). Odds ratio (OR) with 95% CI was determined. A p-value < 0.05 was considered statistically significant.

## Results

### Genotype distributions

Genotype distributions and allele frequencies of rs1801260, rs11932595 and rs4580704 polymorphisms were in Hardy-Weinberg equilibrium and are summarized in Table 1. No significant differences were reported for genotype distribution and allele frequency between women and men.

**Table 1:** Genotype distributions and allele frequencies of the investigated polymorphism.

Genotype	Allele	All	Women	Men	p
**rs1801260**		**n** = **355**	**n** = **257**	**n** = **98**	
AA		168 (47.3)	119 (46.3)	49 (50.0)	0.385
AG		153 (43.1)	110 (42.8)	43 (43.9)	
GG		34 (9.6)	28 (10.9)	6 (6.1)	
	**G**	0.311	0.323	0.281	0.426
**rs11932595**		**n** = **344**	**n** = **247**	**n** = **97**	
AA		114 (33.1)	79 (32.0)	35 (36.1)	0.222
AG		176 (51.2)	124 (50.2)	52 (53.6)	
GG		54 (15.7)	44 (17.8)	10 (10.3)	
	**G**	0.413	0.429	0.371	0.366
**rs4580704**		**n** = **351**	**n** = **255**	**n** = **96**	
CC		149 (42.5)	115 (45.1)	34 (35.4)	0.262
CG		156 (44.4)	108 (42.4)	48 (50.0)	
GG		46 (13.1)	32 (12.5)	14 (14.6)	
	**G**	0.354	0.337	0.396	0.322

### Characteristics of the study participants

The demographic and clinical characteristics of the study participants, according to the genotype, are described in Table 2. Regarding the anthropometric parameters, a significant difference in the prevalence of overweight people based on the presence of the homozygous genotype for the minor allele of rs1801260 (GG: 16.1% vs. AA + AG: 33.7%; p = 0.046) was observed. As of biochemical parameters, after adjustment for medications, subjects homozygous for the minor allele of rs1801260 and rs11932595 showed higher fasting glucose levels (GG: 101.25 (95%CI 93.71–108.79) vs. AA + AG: 92.81 (95%CI 90.33–95.29) mg/dL; p = 0.037 and GG: 99.36 (95%CI 93.22–105.51) vs. AA + AG: 92.51 (95%CI 89.90–95.12) mg/dL; p = 0.044, respectively) compared to those heterozygous and homozygous wild-type. On the other hand, subjects homozygous for the minor allele of rs4580704 showed higher blood triglyceride and LDL-cholesterol levels than those heterozygous and homozygous wild-type (GG: 131.55 (95%CI 116.90–146.20) vs. CC + CG: 111.36 (105.65–117.07) mg/dL; p = 0.012 and GG: 120.81 (95%CI 111.40–130.22) vs. CC + CG: 110.41 (106.74–114.08) mg/dL; p = 0.044). Regarding cognitive and functional abilities, after adjustment for medications, people homozygous for the minor allele of rs1801260 and rs11932595 showed a better score in the clock drawing test (GG: 3.19 (95%CI 1.89–4.49) vs. AA + AG: 4.59 (95%CI 4.15–5.02); p = 0.047 and GG: 3.34 (95%CI 2.28–4.39) vs. AA + AG: 4.61 (4.15–5.07); p = 0.030, respectively) compared to subjects heterozygous and homozygous wild-type, whereas people homozygous for the minor allele of rs1801260 showed a worse score on the geriatric depression scale than heterozygous and homozygous wild-type (GG: 6.32 (95%CI 4.78–7.86) vs. AA + AG: 4.54 (4.03–5.05); p = 0.032). Finally, as regards adherence to the Mediterranean diet a larger number of adherent (with a score ≥34, or median for the study population) were homozygous for the minor allele of rs4580704 compared to heterozygous and homozygous wild-type (GG: 72.7% vs. AA + AG: 56.7%; p = 0.044).

**Table 2:** General characteristics of the Mugello population, according to genotype.

	*rs1801260*			*rs11932595*			*rs4580704*		
	AA + AG (n = 321)	GG (n = 34)	p	AA + AG (n = 290)	GG (n = 54)	p	CC + CG (n = 305)	GG (n = 46)	p
**Anthropometric parameters**									
Female, n (%)	229 (71.3)	28 (82.4)	0.226	203 (70)	44 (81.5)	0.099	223 (73.1)	32 (69.6)	0.723
Age^*^, years	93.01 (92.66–93.37)	92.32 (91.23–93.42)	0.240	93.09 (92.72–93.47)	92.39 (91.52–93.26)	0.146	93.03 (92.67–93.40)	92.13 (91.19–93.07)	0.078
Weight^*^, kg	62.76 (61.34–64.18)	62.00 (57.54–66.46)	0.748	63.01 (61.51–64.52)	61.66 (58.13–65.19)	0.487	62.27 (60.81–63.77)	65.06 (61.28–68.85)	0.177
Height^*^, m	1.58 (1.57–1.59)	1.58 (1.55–1.61)	0.868	1.58 (1.57–1.59)	1.58 (1.55–1.60)	0.908	1.57 (1.56–1.58)	1.60 (1.57–1.62)	0.105
Waist circumference^*^, cm	96.48 (95.08–97.88)	95.38 (91.01–99.74)	0.637	96.67 (95.20–98.15)	95.42 (91.93–98.92)	0.516	96.08 (94.64–97.52)	97.57 (93.84–101.30)	0.464
BMI^*^, kg/m^2^	25.21 (24.70–25.72)	24.84 (23.25–26.44)	0.667	25.30 (24.76–25.84)	24.78 (23.52–26.04)	0.455	25.11 (24.58–25.63)	25.62 (24.24–26.99)	0.494
<18.5, n (%)	18 (6.0)	1 (3.2)	0.527	17 (6.3)	2 (4.1)	0.748	15 (5.2)	4 (10.0)	0.267
18.5–24.9, n (%)	139 (46.3)	20 (64.5)	0.054	124 (45.8)	28 (57.1)	0.162	144 (50.0)	14 (35.0)	0.091
25–29.9, n (%)	101 (33.7)	5 (16.1)	**0**.**046**	91 (33.6)	12 (24.5)	0.246	90 (31.3)	14 (35.0)	0.633
>30, n (%)	42 (14.0)	5 (16.1)	0.530	39 (14.4)	7 (14.3)	0.999	39 (13.5)	8 (20.0)	0.275
**Biochemical parameters**									
Triglycerides^*^, mg/dl	112.80 (107.18–118.42)	123.32 (106.23–140.40)	0.251	112.16 (106.32–118.01)	117.49 (103.75–131.24)	0.484	111.36 (105.65–117.07)	131.55 (116.90–146.20)	**0**.**012**
Total cholesterol^*^, mg/dl	192.07 (187.51–196.64)	198.96 (185.08–212.83)	0.354	193.00 (188.17–197.83)	193.30 (181.93–204.67)	0.962	191.13 (186.50–195.77)	203.76 (191.87–215.65)	0.052
LDL cholesterol^*^, mg/dl	111.73 (108.13–115.34)	114.30 (103.34–125.26)	0.663	112.83 (109.03–116.63)	109.02 (100.08–117.95)	0.440	110.41 (106.74–114.08)	120.81 (111.40–130.22)	**0**.**044**
HDL cholesterol^*^, mg/dl	57.78 (55.89–59.66)	59.99 (54.27–65.72)	0.469	57.74 (55.76–59.72)	60.79 (56.13–65.45)	0.237	58.45 (56.53–60.38)	56.63 (51.69–61.56)	0.498
Fasting glucose^*^, g/L	92.81 (90.33–95.29)	101.25 (93.71–108.79)	**0**.**037**	92.51 (89.90–95.12)	99.36 (93.22–105.51)	**0**.**044**	94.43 (91.86–96.99)	89.14 (82.51–95.77)	0.145
**Blood pressure**									
Systolic blood pressure^*^, mmHg	134.03 (131.84–136.21)	132.63 (125.83–139.43)	0.701	133.34 (131.11–135.56)	134.07 (128.79–139.35)	0.802	134.21 (131.98–136.43)	130.26 (124.43–136.10)	0.215
Diastolic blood pressure^*^, mmHg	72.11 (71.10–73.12)	73.44 (70.29–76.59)	0.430	71.89 (70.85–72.95)	74.10 (71.60–76.61)	0.112	72.23 (71.19–73.26)	71.34 (68.63–74.06)	0.550
**Sleep pattern**									
PSQI^*^, score	7.19 (6.79–7.59)	6.91 (5.64–8.17)	0.672	7.16 (6.73–7.59)	7.11 (6.15–8.07)	0.923	7.15 (6.74–7.56)	7.41 (6.27–8.54)	0.675
Time in bed^*^, min/day	608.24 (584.32–632.16)	654.95 (581.77–728.14)	0.232	612.61 (586.92–638.29)	623.80 (563.43–684.18)	0.736	617.54 (592.51–642.57)	584.31 (523.90–644.71)	0.317
Sleep duration^*^, min/day	495.98 (474.52–517.44)	536.66 (471.00–602.31)	0.246	499.13 (475.96–522.30)	502.20 (447.74–556.66)	0.918	505.87 (483.55–528.18)	462.09 (408.23–515.95)	0.140
Time to fall asleep^*^, min/day	112.26 (100.66–123.86)	118.30 (82.80–153.79)	0.750	113.48 (100.98–125.98)	121.60 (92.21–150.99)	0.616	111.67 (99.60–123.74)	122.22 (93.09–151.34)	0.510
**Cognitive and functional abilities**									
Clock drawing test^*^, score	4.59 (4.15–5.02)	3.19 (1.89–4.49)	**0**.**047**	4.61 (4.15–5.07)	3.34 (2.28–4.39)	**0**.**030**	4.48 (4.03–4.92)	4.47 (3.27–5.66)	0.989
GDS^*^, score	4.54 (4.03–5.05)	6.32 (4.78–7.86)	**0**.**032**	4.53 (3.99–5.07)	5.62 (4.36–6.88)	0.119	4.72 (4.20–5.23)	4.50 (3.12–5.88)	0.774
MMSE^*^, score	18.92 (17.93–19.90)	20.82 (17.81–23.83)	0.238	18.83 (17.79–19.87)	20.42 (18.02–22.82)	0.232	19.32 (18.32–20.32)	17.97 (15.33–20.61)	0.348
BADL^*^, score	2.32 (2.06–2.58)	2.39 (1.60–3.19)	0.861	2.38 (2.11–2.66)	2.12 (1.48–2.75)	0.447	2.39 (2.12–2.65)	2.00 (1.32–2.68)	0.299
IADL^*^, score	4.13 (3.76–4.51)	4.32 (3.19–5.46)	0.756	4.17 (3.77–4.57)	4.06 (3.14–4.98)	0.830	4.21 (3.82–4.59)	3.77 (2.76–4.77)	0.423
Frailty index^*^, score	0.21 (0.19–0.22)	0.20 (0,16–0.24)	0.642	0.21 (0.19–0.22)	0.22 (0.19–0.25)	0.345	0.21 (0.20–0.22)	0.21 (0.18–0.24)	0.945
**Dietary evaluation**									
Med Diet Score^*^, mean ± SD	34.08 (33.66–34.49)	34.35 (33.07–35.63)	0.685	34.08 (33.64–34.52)	34.13 (33.12–35.14)	0.929	33.98 (33.55–34.40)	35.05 (33.93–36.17)	0.081
Adherence MD (≥34 score), n (%)	185 (58.0)	22 (64.7)	0.450	166 (57.6)	33 (61.1)	0.635	173 (56.7)	32 (72.7)	**0**.**044**

^*^Mean ± standard deviation.

### Haplotype analysis

**Figure 1:**
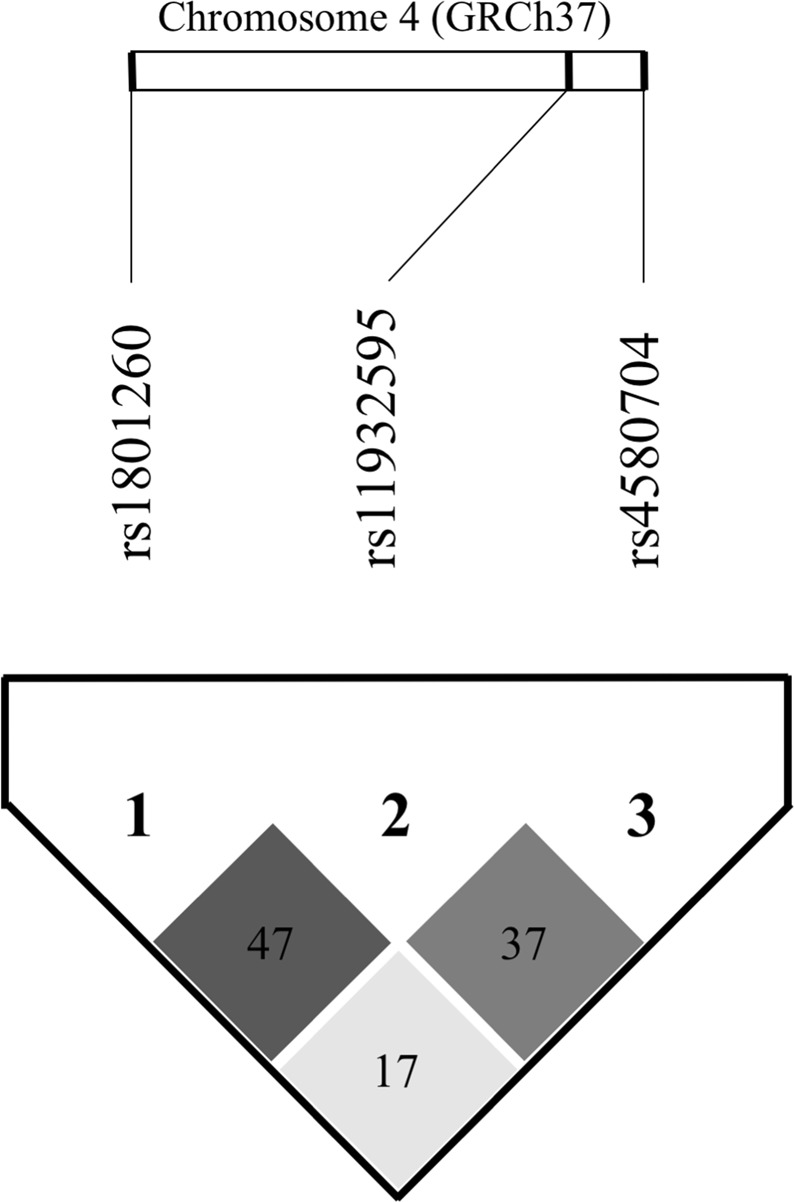
Linkage disequilibrium (LD) plot between the three clock circadian regulator (CLOCK) gene polymorphisms. Black indicates a strong LD (r^2^ = 1.0), white indicates no LD (r^2^ = 0), and gray shading indicates intermediate LD. The numbers inside the diamonds represent r^2^ values × 100.

Figure 1 shows the LD plot among the three polymorphisms of the CLOCK gene. Haplotype reconstruction analysis for the CLOCK gene indicated that the haplotypes of AAC, AAG, GGC and AGC (rs1801260 – rs11932595 – rs4580704) had frequencies of 30.4%, 24.8%, 22.4% and 22.3% respectively. To test any possible association between haplotypes and the tested variables, a logistic regression analysis adjusted for possible confounding factors (e.g. age, sex, BMI and medications) was performed (Supplementary Table 1). The AAG haplotype was associated with a higher risk of overweight (OR = 1.816, 95%CI 1.169–2.821; p = 0.008), hypertriglyceridemia (OR = 1.857, 95%CI 1.029–3.351; p = 0.040), and hypercholesterolemia (OR = 1.663, 95%CI 1.034–2.674; p = 0.036). The GGC haplotype was associated with lower risk of hyperglycemia, comparing people with higher fasting glucose levels (ie >110) to people with low fasting glucose levels (ie ≤110) (OR = 0.426, 95% CI 0.205–0.884; p = 0.022). Furthermore, categorizing people based on scores obtained in PSQI (>7, ie worsening of the sleep pattern compared to ≤7, ie better sleep pattern) and MMSE (>22, or better score vs. ≤22, or worse score), the GGC haplotype was associated with a better sleep pattern (OR = 0.419 95% CI 0.254–0.692; p = 0.001) and with a better score at the MMSE (OR = 0.530, 95% CI 0.327–0.861; p = 0.010). In addition, subjects were classified in depressed and non-depressed based on the GDS score (scores ≥ 5 were considered indicative of depressive status, whereas scores <5 were considered indicative of non-depressive state): the AGC haplotype determined a lower risk of depression (OR = 0.552, 95% CI 0.328–0.931; p = 0.026). Finally, considering adherent those who scored ≥34 points in the MedDiet Score and non-adherent individuals who scored <34 point (ie the median score obtained in the entire study population), the AAC haplotype was associated with a lower degree of adherence to the Mediterranean diet (OR = 1.735, 95% CI 1.061–2.836; p = 0.028).

## Discussion

This is the first study that investigated the role of some *CLOCK* gene SNPs on the quality of aging in a population of very elderly subjects. In the context of the Mugello Study, an epidemiological study aimed at investigating the quality of aging in a cohort of nonagenarians, we were able to demonstrate that some SNPs and the relative haplotypes of the *CLOCK* gene are associated with several traditional cardiovascular risk factors, as with cognitive status, the sleep pattern and adherence to the Mediterranean diet, possibly hypothesizing a role of this gene in the longevity process.

Aging is a physiological process that determines important changes in organ functions and biological variables. One of the most significant alterations includes changes in the output rhythms of the circadian clock because many physiological variables, including those involved in the metabolism are important to ensure that development, survival, and reproduction remain synchronized with environmental changes along the scale of 24-hour^19^. As a result, older individuals typically exhibit a physiological decline in circadian rhythms with many health consequences^20^. The mechanisms underlying these changes are largely unknown, but beyond the changes in the neurochemical and electrophysiological output of the central nervous system, a possible association with some variations of the *CLOCK* gene was also reported^20^. The *CLOCK* is a protein coded by the *CLOCK* gene that regulates the circadian rhythm, so it has been reported that some genetic variations in the gene coding for the *CLOCK* protein can influence the circadian rhythm^21^. Recently, some studies have highlighted a role of the *CLOCK* gene on some aspects of the longevity process, suggesting a possible link between this gene and aging^19–21^.

In the present study, we found an association between some *CLOCK* gene polymorphisms and traditional cardiovascular risk factors such as overweight, glycemia, LDL-cholesterol and triglycerides. To date, cardiovascular diseases are the leading cause of morbidity and mortality even in the elderly, as the quality of aging is extremely affected by the concomitant presence of a cardiovascular disease or a higher cardiovascular risk profile. As already reported by other studies, we confirmed that one of the investigated polymorphism, SNP rs1801260, is related to a reduced risk of overweight, probably mediated by the well-known association of the *CLOCK* gene on the sleep pattern, which in turn predisposes to obesity^8,22^. This was also confirmed by the haplotype analysis, in which it emerged that the AAG haplotype was associated with a lower risk of overweight and that the GGC haplotype was associated with a better sleep pattern.

Furthermore, we have also shown that other gene polymorphisms, namely rs1801260 and rs11932595, are associated with higher levels of fasting glucose and that the GGC (rs1801260-rs11932595-rs4580704) haplotype is related to a lower risk of hyperglycemia. These results can be explained by the numerous influences that these SNPs have on some metabolic processes such as glucose metabolism. In fact, most of the major *CLOCK* genes are expressed in human pancreatic islets, where insulin is rhythmically secreted, and this regulation contributes to glucose homeostasis^23^. Thus, it can be postulated that the effects of the *CLOCK* gene on glucose metabolism in the peripheral organs may be a mechanism involved in the development of hyperglycemia. Next, we also found an association between rs4580704 SNP and higher triglyceride and LDL-cholesterol levels, as well as between AAG haplotype and both higher triglyceride and total cholesterol levels. Many years ago, human and rodent studies^24,25^ have clearly shown that plasma lipids oscillate following a circadian pattern, and it has recently demonstrated that the endogenous circadian clock is the main regulator of these fluctuations in the plasma lipid profile, thus hypothesizing a potential involvement of genetic variations of the *CLOCK* gene^26^.

In addition to cardiovascular risk factors, we were also able to show significant associations between the studied SNPs, their relative haplotypes, and other age-related parameters such as cognitive function, depressive state and quality of diet. In line with previous findings^27^, we found that the subjects homozygous for the minor allele (GG) of the SNP rs1801260 have worse scores on the depression geriatric scale, thus possibly predisposing more significantly to a depressive state. This was also confirmed by the haplotype analysis, in which it emerged that the AGC haplotype was associated with a lower risk of depression. This association can be explained by that fact that depression, like many other psychiatric disorders, is characterized by anomalies of the circadian rhythm, including disturbed sleep/wake cycles and by the evidence connecting the circadian clock and the stress response systems^28^. The circadian *CLOCK* gene regulates physiological sensitivity to the rhythmic release of glucocorticoids, which, in turn, have mutual effects on the protein, as stressful life events or increased vulnerability to stress are risk factors for multiple psychiatric disorders including major depressive disorder, modulation of the stress response can represent a common mechanism by which circadian *CLOCK* gene influences psychiatric diseases including depression^28^.

On the other hand, we were able to show, for the first time, that in this particular cohort of elderly subjects, the SNPs rs1801260 and rs11932595 of the *CLOCK* gene are associated with a better score at the clock drawing test, suggesting a potential beneficial role of the G allele of these SNPs with respect to visuo-constructional skills and to abstract thinking. This can be explained by the fact that, as previously discussed, circadian clock variations lead to an altered stress response, in which the glucocorticoids play an important role. The increased release of glucocorticoids in response to acute stress, due to an alteration of the circadian clock, leads to increased alertness, to the mobilization of glucose and fatty acids, and to a better memory formation^28^. This was also confirmed by the haplotype analysis that showed an association between GGC haplotype and a better score at the MMSE.

Finally, with regard to adherence to the Mediterranean diet, although the MedDiet score was similar among the tested SNPs’ genotypes, we found a greater number of adherents who were homozygous for the rs4580704 minor allele compared to heterozygous and homozygous wild-type. This was also confirmed by the haplotype analysis that showed that AAC haplotype was associated with a low degree of adherence to the Mediterranean diet. Very recently we reported in the same group of subjects that a greater degree of adherence to Mediterranean diet was associated with a reduced risk of depression^29^, so we can hypothesize a role of the *CLOCK* gene also in the modulation of the depressive status through the modification of the quality of the diet.

This paper presents strengths and limitations. The strengths are the peculiar population of evaluated nonagenarians and the parameters analyzed. No other study, to the best of our knowledge, has previously studied the possible influence of *CLOCK* gene SNPs on the quality of aging in this particular group of subjects. On the other hand, this study has some limitations that must to be considered. First of all, due to the fact that we considered a peculiar and valuable cohort of nonagenarians, the number of subjects analyzed was not so large. Second, adherence to prescribed medications was not evaluated, so possibly influencing the results of the study. In addition, the results of this study were based on a cross-sectional analysis, which is commonly considered less robust than longitudinal analyses. However, this limitation is quite common in studies involving the older old as lethality heavily affects the availability of participants for follow-ups.

In conclusion, the CLOCK gene seems to have a multi-function that globally and independently of the physiological decay is able to regulate some metabolic and psychiatric parameters in nonagenarians. Indeed, the present study suggests an association between the *CLOCK* gene polymorphisms and their derived haplotypes with different cardiovascular risk factors, as well as with the cognitive state, sleep pattern and the adherence to the Mediterranean diet in a cohort of very old subjects. These results suggest that the presence of some SNPs, added to the physiological desynchronization of the typical circadian rhythm of the elderly, can have a serious impact on general health, possibly hypothesizing a role of this gene on the quality of aging.

## Supplementary information

Supplementary Table 1

## Data Availability

The datasets analyzed during the current study are available from the corresponding author on reasonable request.

## References

[1] Newgard CB, Sharpless NE (2013). Coming of age: molecular drivers of aging and therapeutic opportunities. J Clin Invest..

[2] Cazaly E, Charlesworth J, Dickinson JL, Holloway AF (2015). Genetic Determinants of Epigenetic Patterns: Providing Insight into Disease. Molecular Medicine..

[3] Bass J, Takahashi JS (2011). Circadian integration of metabolism and energetics. Science..

[4] Valladares M, Obregón AM, Chaput JP (2015). Association between genetic variants of the clock gene and obesity and sleep duration. J Physiol Biochem..

[5] McCarthy MJ, Welsh DK (2012). Cellular circadian clocks in mood disorders. J Biol Rhythms..

[6] Kelleher FC, Rao A, Maguire A (2014). Circadian molecular clocks and cancer. Cancer Lett..

[7] Antoch MP (2008). Disruption of the circadian clock due to the Clock mutation has discrete effects on aging and carcinogenesis. Cell Cycle..

[8] Galbete C (2012). Physical Activity and Sex Modulate Obesity Risk Linked to 3111T/C Gene Variant of the *CLOCK* Gene in an Elderly Population: The SUN Project. Chronobiol Int..

[9] Molino-Lova R (2013). Mugello Study Working Group. The Mugello study, a survey of nonagenarians living in Tuscany: design, methods and participants’ general characteristics. Eur J Intern Med..

[10] Mollayeva T (2016). The Pittsburgh Sleep Quality Index as a screening tool for sleep dysfunction in clinical and non-clinical samples: a systematic review and meta- analysis. Sleep Med Rev..

[11] Sheikh JI, Yesavage JA (1986). Geriatric Depression Scale (GDS): recent evidence and development of a shorter version. Clin Gerontol..

[12] Folstein MF, Folstein SE, Mchugh PR (1975). “Mini-mental state”. A practical method for grading the cognitive state of patients for the clinician. J Psychiatr Res..

[13] Lam LC (1998). Clock-face drawing, reading and setting tests in the screening of dementia in Chinese elderly adults. J Gerontol B Psychol Sci Soc Sci..

[14] Katz S, Ford AB, Moskowitz RW, Jackson BA, Jaffe MW (1963). Studies of illness in the aged. the index of adl: a standardized measure of biological and psychosocial function. JAMA..

[15] Lawton MP, Brody EM (1969). Assessment of older people: self-maintaining and instrumental activities of daily living. Gerontologist..

[16] Kim S, Welsh DA, Cherry KE, Myers L, Jazwinski SM (2013). Association of healthy aging with parental longevity. AGE..

[17] Panagiotakos DB, Pitsavos C, Arvaniti F, Stefanadis C (2007). Adherence to the Mediterranean food pattern predicts the prevalence of hypertension, hypercholesterolemia, diabetes and obesity, among healthy adults; the accuracy of the MedDietScore. Prev Med..

[18] Livak KJ (1999). Allelic discrimination using fluorogenic probes and the 5′ nuclease assay. Genet Anal..

[19] Liu F, Chang HC (2017). Physiological links of circadian clock and biological clock of aging. Protein Cell..

[20] Froy O (2011). Circadian Rhythms, Aging, and Life Span in Mammals. Physiology (Bethesda)..

[21] Gibson EM, Williams WP, Kriegsfeld LJ (2009). Aging in the circadian system: considerations for health, disease prevention and longevity. Exp Gerontol..

[22] Tortorella A, Monteleone P, Martiadis V, Perris F, Maj M (2007). The 3111T/C Polymorphism of the CLOCK Gene Confers a Predisposition to a Lifetime Lower Body Weight in Patients with Anorexia Nervosa and Bulimia Nervosa: A Preliminary Study. Am J Med Genet..

[23] Stamenkovic JA (2012). Regulation of core clock genes in human islets. Metabolism..

[24] Schlierf G, Dorow E (1973). Diurnal patterns of triglycerides, free fatty acids, blood sugar, and insulin during carbohydrate-induction in man and their modification by nocturnal suppression of lipolysis. J Clin Invest..

[25] Fukagawa K, Gou HM, Wolf R, Tso P (1994). Circadian rhythm of serum and lymph apolipoprotein AIV in ad libitum-fed and fasted rats. Am. J. Physiol..

[26] Dallmann R, Viola AU, Tarokh L, Cajochen C, Brown SA (2012). The human circadian metabolome. Proc. Natl. Acad. Sci. USA.

[27] Benedetti F (2015). Effects of CLOCK gene variants and early stress on hopelessness and suicide in bipolar depression. Chronobiol Int..

[28] Landgraf D, McCarthy MJ, Welsh DK (2014). Circadian clock and stress interactions in the molecular biology of psychiatric disorders. Curr Psychiatry Rep..

[29] Pagliai G (2018). Mediterranean diet, food consumption and risk of late-life depression: The Mugello Study. J Nutr Health Aging..

