# Supplementary material for: CLOCK gene polymorphisms and quality of aging in a cohort of nonagenarians – The MUGELLO Study
Source: Sci Rep. 2019 Feb 6;9:1472. doi: 10.1038/s41598-018-37992-8 (PMC6365537; doi:10.1038/s41598-018-37992-8)
Supplement: Supplementary file 1 — Supplementary Table 1 [file 41598_2018_37992_MOESM1_ESM.pdf]

CLOCK gene polymorphisms and quality of aging in a cohort of nonagenarians – The MUGELLO Study

Giuditta PAGLIAI [1*], MSc
Francesco SOFI [1,2], MD, PhD
Monica DINU [1], MSc, PhD
Elena STICCHI [1,3], MSc, PhD
Federica VANNETTI [2], PhD
Raffaello MOLINO LOVA [2], MD
José Marìa ORDOVÀS [4], PhD
Anna Maria GORI [1,3], MSc
Rossella MARCUCCI [1,3], MD, PhD
Betti GIUSTI [1,3], MSc, PhD
Claudio MACCHI [2], MD

[1] Department of Experimental and Clinical Medicine, University of Florence, Italy; [2] Don Carlo Gnocchi Foundation Florence, Onlus IRCCS, Florence, Italy; [3] Atherothrombotic Unit, Careggi University Hospital, Florence; [4] Jean Mayer US Department of Agriculture Human Nutrition Research Center on Aging, Tufts University School of Medicine, Boston, MA, USA

Supplementary Table 1. Logistic regression analysis of cardiovascular risk factors, cognitive and functional parameters according to haplotype.

Risk factors	AAC	p	AAG	p	GGC	p	AGC	p
**Overweight**	1.043 (0.651-1.671)	0.860	1.816 (1.169-2.821)	**0.008**	0.939 (0.609-1.448)	0.776	0.966 (0.627-1.490)	0.877
**High waist circumference**	1.174 (0.717-1.924)	0.524	1.232 (0.779-1.948)	0.373	1.048 (0.664-1.652)	0.841	0.972 (0.616-1.533)	0.903
**Hypertriglyceridemia**	0.640 (0.355-1.154)	0.138	1.857 (1.029-3.351)	**0.040**	0.682 (0.387-1.201)	0.185	0.778 (0.442-1.369)	0.385
**High total cholesterol**	0.994 (0.598-1.654)	0.982	1.663 (1.034-2.674)	**0.036**	1.068 (0.671-1.700)	0.781	1.050 (0.658-1.674)	0.838
**High LDL cholesterol**	0.866 (0.514-1.459)	0.588	1.593 (0.985-2.576)	0.058	1.237 (0.768-1.991)	0.381	1.146 (0.711-1.846)	0.577
**Low HDL cholesterol**	0.650 (0.311-1.359)	0.252	0.897 (0.453-1.773)	0.754	0.611 (0.306-1.223)	0.164	0.684 (0.345-1.360)	0.279
**Hyperglycemia**	1.455 (0.646-3.276)	0.366	1.041 (0.520-2.086)	0.910	0.426 (0.205-0.884)	**0.022**	0.686 (0.339-1.389)	0.295
**Hypertension**	1.207 (0.673-2.165)	0.528	1.429 (0.848-2.408)	0.180	1.276 (0.759-2.145)	0.358	1.361 (0.807-2.293)	0.248
**Worse PSQI**	0.921 (0.542-1.566)	0.763	0.723 (0.443-1.181)	0.195	0.419 (0.254-0.692)	**0.001**	0.946 (0.580-1.544)	0.946
**Worse CDT**	1.406 (0.845-2.339)	0.190	1.303 (0.821-2.068)	0.261	0.741 (0.467-1.174)	0.202	1.194 (0.755-1.888)	0.449
**Worse GDS**	0.612 (0.350-1.070)	0.085	0.605 (0.360-1.017)	0.058	0.714 (0.429-1.188)	0.195	0.552 (0.328-0.931)	**0.026**
**Worse MMSE**	1.162 (0.694-1.947)	0.568	1.466 (0.906-2.373)	0.120	0.530 (0.327-0.861)	**0.010**	0.913 (0.567-1.473)	0.710
**Worse BADL**	1.426 (0.857-2.373)	0.172	0.931 (0.584-1.482)	0.763	0.722 (0.453-1.150)	0.171	1.440 (0.904-2.292)	0.125
**Worse IADL**	1.150 (0.698-1.895)	0.584	0.860 (0.541-1.366)	0.522	0.855 (0.539-1.356)	0.505	1.292 (0.814-2.049)	0.277
**Worse Frailty index**	0.623 (0.324-1.198)	0.156	1.012 (0.555-1.846)	0.969	1.054 (0.578-1.920)	0.864	0.681 (0.370-1.254)	0.271
**Worse Med Diet Score**	1.735 (1.061-2.836)	**0.028**	1.033 (0.664-1.605)	0.886	1.141 (0.736-1.769)	0.555	1.040 (0.671-1.612)	0.862
